# A New Approach to Reduce Uncertainties in Space Radiation Cancer Risk Predictions

**DOI:** 10.1371/journal.pone.0120717

**Published:** 2015-03-19

**Authors:** Francis A. Cucinotta

**Affiliations:** Department of Health Physics and Diagnostic Sciences, University of Nevada, Las Vegas, Nevada, United States of America; Belgian Nuclear Research Centre SCK•CEN, BELGIUM

## Abstract

The prediction of space radiation induced cancer risk carries large uncertainties with two of the largest uncertainties being radiation quality and dose-rate effects. In risk models the ratio of the quality factor (QF) to the dose and dose-rate reduction effectiveness factor (DDREF) parameter is used to scale organ doses for cosmic ray proton and high charge and energy (HZE) particles to a hazard rate for γ-rays derived from human epidemiology data. In previous work, particle track structure concepts were used to formulate a space radiation QF function that is dependent on particle charge number Z, and kinetic energy per atomic mass unit, E. QF uncertainties where represented by subjective probability distribution functions (PDF) for the three QF parameters that described its maximum value and shape parameters for Z and E dependences. Here I report on an analysis of a maximum QF parameter and its uncertainty using mouse tumor induction data. Because experimental data for risks at low doses of γ-rays are highly uncertain which impacts estimates of maximum values of relative biological effectiveness (RBE_max_), I developed an alternate QF model, denoted QF_γAcute_ where QFs are defined relative to higher acute γ-ray doses (0.5 to 3 Gy). The alternate model reduces the dependence of risk projections on the DDREF, however a DDREF is still needed for risk estimates for high-energy protons and other primary or secondary sparsely ionizing space radiation components. Risk projections (upper confidence levels (CL)) for space missions show a reduction of about 40% (CL∼50%) using the QF_γAcute_ model compared the QFs based on RBE_max_ and about 25% (CL∼35%) compared to previous estimates. In addition, I discuss how a possible qualitative difference leading to increased tumor lethality for HZE particles compared to low LET radiation and background tumors remains a large uncertainty in risk estimates.

## Introduction

Fatal cancer risks are a concern for astronauts on long-term space exploration missions due to exposures to galactic cosmic rays (GCR) and secondary radiation—made up predominantly of high-energy protons, high-energy and charge (HZE) nuclei and neutrons, and possible solar particle events (SPEs)—comprised largely of low- to medium-energy protons. NASA has followed recommendations from the National Council of Radiation Protection and Measurements (NCRP) for setting radiation dose limits [1, 2]. The uncertainties in estimating space radiation risks have been recognized by several reports from the NCRP [1–4] and National Research Council (NRC) [5–7]. The uncertainty is largely due to the lack of information on the radiobiology of HZE particles that produce both quantitative and qualitative differences in biological effects compared to γ-rays or x rays, and for which no human data is available. This uncertainty led the NCRP to recommend that methods used for space missions in low Earth orbit (LEO) are not of sufficient accuracy for long-term exposure (>30 d) to GCR [2]. In the face of the obstacles of large radiobiological uncertainties while needing to support current missions and future mission planning, NASA developed an approach to estimate the 95% confidence intervals in cancer risk estimates using methods developed by the NCRP for low LET radiation [8] that was extended to the space radiation exposures [9–11]. This approach formed the basis for the current NASA radiation dose limits [12] and was supported by a NRC review [6, 7].

Two of the largest uncertainties for space radiation risk estimates are the radiation quality factor (QF) function and the dose and dose-rate reduction effectiveness factor (DDREF). In a recent report particle track structure descriptions of microscopic energy deposition was used to develop a new space radiation QF [10], which led to improvements [6] over the previous linear energy transfer (LET) dependent QFs [2]. Other smaller uncertainties include those related to space radiation environments and organ dose assessments, epidemiology data, and the extrapolation of these data to a model population. In addition, there are uncertainties related to assumptions made within model. The two most critical of these appears to be the use of a linear dose response model, which is challenged by observations of non-targeted effects (NTE) at low doses for high LET radiation [13–15], and the implicit assumption that high LET radiation induced tumors are of similar lethality as low LET induced or background tumors [16]. Other considerations include non-cancer risks that could increase risk of exposure induced death (REID) estimates, including late effects related to circulatory and central nervous system diseases [3,11,17], and the development of approaches for individual based risk assessment [18].

Estimates of maximum relative biological effectiveness (RBE_max_) defined by the ratio of initial linear slopes determined at low dose and dose-rate for particles to γ-rays are used in radiation protection to assign values of QFs. The QF multiplies the organ specific absorbed dose to define an organ dose equivalent for particles relative to a reference radiation, which is ^60^Co γ-rays. Values of RBE_max_ are highly dependent on the reference radiation used and their responses at low dose and dose-rates. The large values of RBE_max_ found in many experiments can be attributed in-part to the ineffectiveness of low doses or low dose-rates of γ-rays. In addition, not all experiments have used either low dose-rates (<0.1 Gy/hr) or low doses (<0.25 Gy) of γ-rays thus precluding RBE_max_ estimates. A linear-quadratic dose response model can be fit to acute γ-ray experimental data for tumor induction if a sufficient range of doses were considered, allowing for an estimate of RBE_max_ by assuming the linear component represents the low dose-rate response. However this introduces an additional uncertainty due to possible differences in linear slopes derived from acute compared to low dose-rate experiments.

High LET radiation generally shows a small or no dose-rate dependence in contrast to low LET radiation where low dose-rate can significantly reduce effects. For considering risks from fission neutron exposures, Edwards [19] discussed the direct use of RBE from higher doses and dose-rates thereby avoiding the use of low dose-rate data for γ-rays and its associated uncertainties. Space radiation is a complicated mixture of high and low LET radiation and the description of dose-rate modifiers cannot be entirely avoided. In this paper I develop a new model of QF’s which effectively interpolates the QF between the high LET case where the lack of a dose-rate effect can be reasonably assumed to the low LET case where a DDREF should be applied. My approach is based on the functional form of the NASA QF which is based on a parametric track structure model [6, 20] that effectively divides a particle track between high and low ionization density regions.

For high LET radiation the dose response for tumor induction is expected to be linear over the dose range from about 0.1 to 0.4 Gy with little dependence on dose-rate, especially at lower doses [21–30], however observed tumor dose responses often display a downward curvature as dose is increased. Also, there is the possibility of supra-linear responses at the most relevant doses (<0.1 Gy) due to non-targeted effects [13]. For the purposes of this report I will assume a linear dose response model at high LET is approximately correct for RBE estimates, and ignore the possibility of a non-linear response at low doses.

A second new aspect of the current work is to use the maximum likelihood method to estimate the uncertainty in a key parameter in the NASA QF model, denoted as the ratio Σ_0_ / *α*_*γ*_, which is related to the largest QF value that occurs for any mono-energetic particle of atomic number, Z and kinetic energy per nucleon, E. This revised approach avoids the previous subjective estimate [10] which was based in-part on endpoints in cell culture models, which are likely deficient in representing cancer risk. Leukemia risks were found to be much smaller compared to solid cancers for high LET radiation in the previous reports [6, 10]. The previous approach [10] to leukemia risk estimates will be followed herein. A further development is to use probability distribution functions (PDF’s) for the uncertainty in the DDREF parameter based on the Bayesian approach using the BEIR VII Report on the PDF for the Japanese Life Span Study (LSS) combined with DDREF values from the same mouse tumor induction studies used for QF estimates. This approach is viewed as superior compared to the use of unmatched data sets because of the possible correlation between RBE and DDREF estimates. This new estimate is then used to update the NSCR-2012 model, which is denoted as NSCR-2014, and comparisons of the different approaches made for long-term space missions near solar minimum. The QF model based on acute γ-ray dose responses for solid tumor induction denoted, QF_γAcute_ is shown to reduce upper confidence intervals of space mission risk predictions by about 50% compared to QF’s based on RBE_max_.

## Methods

### Cancer Risk Projection Model

The instantaneous cancer incidence or mortality rates, λ_I_ and λ_M_, respectively, are modeled as functions of the tissue averaged absorbed dose *D*_*T*_, or dose-rate *D*_*Tr*_, gender, age at exposure *a*_*E*_, and attained age *a* or latency *L*, which is the time after exposure *L = a-a*_*E*_. The λ_I_ (or λ_M_) is a sum over rates for each tissue that contributes to cancer risk, λ_IT_ (or λ_MT_). These dependencies vary for each cancer type that could be increased by radiation exposure. The total risk of exposure induced cancer (REIC) is calculated by folding the instantaneous radiation cancer incidence-rate with the probability of surviving to time *t*, which is given by the survival function *S*_*0*_ *(t)* for the background population times the probability for radiation cancer death at previous time, summing over one or more space mission exposures, and then integrating over the remainder of a lifetime [16, 29]:

$$\begin{array}{ccc} \mathrm{REIC}\left(a_{E},D_{T}\right) & = & \sum_{j=1}^{N_{m}}\int_{a_{Ej}}dt\;\lambda_{Ij}\left(a_{E_{j}},t,D_{Tj}\right)S_{0}\left(t\right)e^{-\sum_{k=1}^{N_{m}}\;\int_{a_{{}_{E}}}^{t}dz\;\lambda_{M_{k}}\left(a_{E_{k}},z,D_{T}{}_{k}\right)} \end{array}$$ (1)

where z is the dummy integration variable. In Equation (1), N_m_ is the number of missions (exposures), and for each exposure, j, there is a minimum latency of 5-years for solid cancers, and 2-years for leukemia assumed. Tissue specific REIC estimates are similar to Equation (1) using the single term from λ_I_ of interest. The equation for REID estimates is similar to Equation (1) with the incidence rate replaced by the mortality rate (defined below).

After adjustment for low dose and dose-rates through introduction of the dose and dose-rate effectiveness factor (DDREF) and radiation quality through the space radiation QF, the tissue-specific cancer incidence rate for an organ absorbed dose, *D*_*T*_, is written as a weighted average of the multiplicative and additive transfer models, denoted as a mixture model:

$$\begin{array}{ccc} \lambda_{IT}\left(a_{E},a,H_{T}\right) & = & \left[v_{T}ERR_{T}\left(a_{E},a\right)\lambda_{0IT}\left(a\right)+\left(1-v_{T}\right)EAR_{T}\left(a_{E},a\right)\right]\frac{QFxD_{T}}{DDREF} \end{array}$$ (2)

where *v*_*T*_ is the tissue-specific transfer model weight, *λ*_*0IT*_ is the tissue-specific cancer incidence rate in the reference population, and where *ERR*_*T*_ and *EAR*_*T*_ are the tissue specific excess relative risk and excess additive risk per Sievert, respectively, that are described in other reports [29–31].

The tissue specific rates for cancer mortality *λ*_*MT*_ are modeled following the BEIR VII report [30] whereby the incidence rate of Equation (2) is scaled by the age, sex, and tissue specific ratio of rates for mortality to incidence in the population under study:

$$\begin{array}{ccc} \lambda_{MT}\left(a_{E},a,H_{T}\right) & = & \frac{\lambda_{0MT}\left(a\right)}{\lambda_{0IT}\left(a\right)}\lambda_{IT}\left(a_{E},a,H_{T}\right) \end{array}$$ (3)

The U.S. cancer rates from 2011 as represented by the DEVCAN software (Version 6.7.2) available from the Center of Disease Control (CDC) are used in this report [32]. DEVCAN provides age, sex and tissue specific incidence and mortality data to ages 95+, which improves on the extrapolation of cancer rates to older ages (>85 y) used in the NSCR-2012 model [10]. Cancer rates for different races are available as considered in an earlier report [10]. Here we used the U.S. average rates from DEVCAN [32].

### Space Radiation Organ Dose Equivalent

I consider the QF function divided by the DDREF as being made-up of two terms:

$$\begin{array}{ccc} \frac{QF\left(Z,E\right)}{DDREF} & = & \frac{Q_{low}\left(Z,E\right)+Q_{high}\left(Z,E\right)}{DDREF} \end{array}$$ (4)

In Equation (4) Q_high_ and Q_low_ roughly represent the contributions from a particle track acting in high density (track core) or low density modes (track penumbra), respectively with the radiosensitivity parameters described below defining these relative contributions. Secondary electrons produced through ionizations denoted as δ-rays contribute in both regions of a particle track. However energy deposition in bimolecular targets by single δ-rays of higher energy (>10 keV) are more typical of the low density part of the track, while multiple δ-rays of lower energy along with the particle itself interact with biomolecular targets in the high density region of the track close to the particles path (<100 nm). δ-rays in the penumbra can extend laterally for many microns from an HZE particles path. These functions are

$$Q_{low}\left(Z,E\right)\;=\left(1-P\left(Z,E\right)\right)$$ (5)

and

$$\begin{array}{cc} Q_{high}\left(Z,E\right) & =\frac{\left(\Sigma_{0}/\alpha_{\gamma }\right)P\left(Z,E\right)}{LET} \end{array}$$ (6)

where the ratio Ʃ_0_/*α*_*γ*_ is used as a single parameter, and the function originating in the parametric model of Katz [20] is given by,

$$P(Z,E)={\left(1-e^{-Z^{*2}/\kappa \beta^{2}}\right)}^{m}\left(1-e^{-E/E_{TD}}\right)$$ (7)

The second product in Equation (7) represents a so-called thin-down correction for low energy particles (E<1 MeV/u) [10]. The space radiation QF depends on two physical parameters: particle charge number, Z and kinetic energy per atomic mass unit, E. However, a key parameter that describes the density of a particle track is Z*^2^/ β^2^, where Z* is the effective charge number [33] of a particle and β is the particle velocity scaled to the speed of light, is used to simplify the description [10].

In the NSCR-2012 model distinct quality factors for estimating solid cancer and leukemia risk are used [10]. A DDREF is not used in Equation (2) for leukemia risk estimates and instead the linear component of a linear-quadratic dose response model fit to human γ-ray epidemiology data is used. The parameters of the model (Ʃ_0_/*α*_*γ*_, E_TD_, *m*, and *Κ*) listed in Table 1 are estimated based on subjective estimates of results from radiobiology experiments [10].

**Table 1:** Parameters for central estimate of 2012 NASA quality factor (QF) parameters for solid cancer and leukemia risks [10]. *

*Parameter*	*Solid Cancer*	*Leukemia*
***m***	3	3
***k***	550 (1000)	550 (1000)
***Ʃ_0_/α_γ_, μm^2^ Gy***	7000/6.24	1750/6.24
***E_TD._ MeV/u***	0.15	0.15

*Values in parenthesis are distinct values for light ions (Z ≤ 4).

The alternative QF introduced here is to make an assessment of QFs based on RBE’s determined from low dose and dose-rate particle data relative to acute γ-ray from experiments for doses of about 0.5 to 3 Gy denoted as RBE_γAcute_ which was suggested by Edwards [19]. This approach alleviates the need to consider low dose-rate γ-ray experiments for RBE estimates, however for low LET particles a DDREF is still warranted because of their expected reduced effectiveness at low dose-rates compared to acute γ-ray exposures at higher doses. The alternative QF is then written:

$$\begin{array}{cccc} & \frac{QF_{\gamma Acute}(Z,E)}{DDREF} & = & \frac{Q_{low}(Z,E)}{DDREF}+{\bar{Q}}_{high}(Z,E) \end{array}$$ (8)

Where ${\bar{Q}}_{high}$ uses a parameter estimate for Ʃ_0_/*α*_*γ*_ based on estimates of RBE_γAcute_ as described above instead of RBE_max_. The key assumption of the new model given by Equation (8) is that the low ionization density part of a particle track is influenced by dose-rate effects as represented by the first term on the right hand side of Equation (8), while the high ionization density part of a particles track has no dependence on dose-rate as described by the second term on the right-hand side of Equation (8). The QF function shape parameters, *m* and *Κ* are taken as the same as in the NSCR-2012 model [10].

### Estimates of PDF for Ʃ_0_/*α*_*γ*_ and DDREF Parameters

I formed a cumulative distribution function (CDF) for values of RBE_max_ and RBE_γAcute_ based on available experimental data in mice [10, 13, 21–25, 27, 28, 36, 37]. The CDF was then fit using the non-linear regression modules in *Sigmaplot 12*.*1* to a 3-parameter logistics function.

For the DDREF estimate I updated the previous PDF estimates [10] using the Bayesian theory. Here I combined a prior distribution based on the BEIR-VII report estimates of Japanese LSS data [30] with a log-normal distribution to represent the matched mouse tumor induction data used for RBE estimates to form the posterior distribution.

Table 2 lists the experimental data for solid tumors in mice [21–28, 34, 35] that is considered for forming the likelihood function. Listed are tumor and mouse strain specific estimates of RBE_max_, DDREF, and RBE_γAcute_ for fission neutrons and HZE particles which are expected to be near the maximum biological effectiveness as a function of Z and E. For experiments with HZE particles where more than one particle was used for the same tumor type, only the average values of these data is used to avoid the use of redundant information. The question of whether such results are reflective of the highest effective particle types for solid cancer induction is discussed next.

**Table 2:** Relative biological effectiveness (RBE) factors and dose and dose-rate reduction effectiveness factors (DDREF) for solid tumors from mouse experiments.

Tumor Type	Mouse Strain	Sex	Radiation, LET(keV/μm) (Energy, MeV/u)	RBE_max_	DDREF	RBE_γAcute_	References
Harderian Gland*	B6CF1	F	Fe, 180 (600)	39.6±11.5	-	-	[25]
				27	-	-	[24]
				28	2.17 ± 1.1	14	[10,13]
Harderian Gland	B6CF1	F	Ar SOBP**, ~200	27	-	-	[24]
Hepatocellular carcinoma	CBA	M	Fe, 155 (1000)	Not Estimated	-	50.9±9.9	[27]
Hepatocellular carcinoma	C3H/HeNCrl	M	Fe 175 (600)	Not Estimated	-	66.9±41.1	[28]
Hepatocellular carcinoma	C3H/HeNCrl	M	Si, 70 (300)	Not Estimated	-	73.5±46.6	[28]
Lung	BALB/c	F	Fission neutrons	33±12	2.8	11.8	[21–23]
Mammary	BALB/c	F	Fission neutrons	18.5±6	1.9	9.7	[21–23]
Pituitary	RFM	F	Fission neutrons	59±52	2.6	22.5	[21,36]
Harderian Gland	RFM	F	Fission neutrons	36±10	2.5	14.6	[21,36]
All Epithelial	B6CF1	M	Fission neutrons	28.3±4.0	2.3±0.3	12.1±4.5	[37]
Lung	B6CF1	M	Fission neutrons	24.3±4.6	2.2±0.3	11.0±2	[37]
Liver	B6CF1	M	Fission neutrons	39.1±12.1	2.0±0.3	19.3±5.6	[37]
Glandular and Reproductive Organs	B6CF1	M	Fission neutrons	49.3±7.8	4.3±0.3	16.6±5.6	[37]
Harderian Gland	B6CF1	M	Fission neutrons	50.7±10.8	4.7±0.3	12.1±2.9	[37]
All Epithelial	B6CF1	F	Fission neutrons	21.9±3.3	1.7±0.3	11.0±1.6	[37]
Lung	B6CF1	F	Fission neutrons	18.1±4.2	1.8±0.3	10.3±2.2	[37]
Liver	B6CF1	F	Fission neutrons	23.3±11.6	5.9±0.3	4.4±1.6	[37]
Glandular and Reproductive Organs excluding Ovarian	B6CF1	F	Fission neutrons	84.4±20.8	12.2±0.3	7.4±1	[37]
Harderian Gland	B6CF1	F	Fission neutrons	61.9±31.5	8.7±0.3	5.8±1.2	[37]

Estimates of the maximum relative biological effectiveness (RBE) factor, RBE_max_, the tumor specific dose and dose-rate reduction effectiveness factor (DDREF), and RBE_γAcute_ for low dose high charge and energy (HZE) particles and fission neutrons relative to acute γ-rays at moderate to high acute doses (~0.5 to ~3 Gy).

*RBEs were estimated using different approaches in references cited. We used the estimate from a global fit to all radiation types considered [10,13], which is similar to the estimate of Fry et al. [24].

**SOPB is spread-out Bragg peak irradiation.

### Maximum Effective Particle

Published solid tumors studies with fission neutrons or HZE particles with specific values of Z and E do not necessarily reflect the most biologically effective particle type that may occur. For example a hypothetical study with uniform irradiation by mono-energetic protons of low energy (∼ 0.5 MeV) is predicted to be more effective than a ^252^Cf fission neutron source where a broad spectrum of proton energies with small contributions from other recoil particles occurs. In a similar manner, compared to high energy Fe particles a particle of lower Z and E could have a higher biological effectiveness [10]. Using the QF shape parameters, *Κ* and *m*, and published data on spectra [36, 37] and radiation transport codes [38] that predict the charged particle spectra in mice from fission neutrons, the charged particle spectra can be folded with the QF functions described above to find dose-averaged RBE values. The analysis proceeds by folding Equation (4) or alternatively Equation (8) with the energy spectra of charged particles produced by fission neutrons to find the dose average RBE values which are compared to the experimental data. Using this approach, the values for Ʃ_0_/*α*_*γ*_ that agreed well with RBE data for both fission neutrons and Fe particles were found as described below. The uncertainty in this estimate, which includes estimating particle energy spectra in fission neutron exposures, overlaps to a large extent with the uncertainty in the other QF parameter estimates and therefore a new uncertainty parameter is not introduced.

### Applications to Space Mission Assessments

For the application of the model to space mission predictions the energy spectra for each particle type, *j* of LET, *L*_*j*_ *(E)* for each tissue, *T* contributing to cancer risk denoted as *ϕ*_*jT*_ *(E)* is estimated from radiation transport codes [10, 11, 16]. The particle energy spectra are folded with the QF and DDREF factors described above to estimate tissue specific or total REID values [10, 11, 16].

### Sensitivity Study of Increased HZE Particle Tumor Lethality

Studies of tumors in mouse [25, 28, 39–41] by highly ionizing radiation, suggest that solid tumors produced by HZE particles are qualitatively distinct from background tumors or tumors induced by low LET with observations of higher tumor grade, and increased propensity for metastatic tumors. An upper limit on the possibility of higher tumor lethality would be to use REIC estimates for REID estimates on space missions. However this estimate would be too large due to the presence of low LET particles such as protons that make up a significant fraction of space radiation organ doses. To make a more realistic estimate of the effects of an increased lethality the cancer mortality rate is modified as [16]

$$\begin{array}{cccc} & \lambda_{MT} & \approx & \frac{\lambda_{0MT}(a)}{\lambda_{0IT}(a)}\lambda_{I\gamma T}\left\{\sum_{j}\int dE\varphi_{jT}(E)L_{j}(E)(1-P(X_{tr}))+(\Sigma_{0}/\alpha_{\gamma })F_{lethal}\int dX_{tr}\varphi_{T}(X_{tr})P(X_{tr})\right\} \end{array}$$ (9)

where the second term in Equation (9) is increased by a tumor lethality fraction, F_lethal._ The second term in Equation (9) has been reduced to be independent of the particle type, *j*, using the variable X_tr_ = Z*^2^/β^2^ as described previously [10]. The first term in Equation (9) dominates for low LET radiation and is not altered under the considerations of increased tumor lethality for highly ionizing radiation. For the sensitivity study of F_lethal_, I considered a PDF to represent the uncertainty in the increased lethality for HZE particles and secondary charged particles from neutrons. The PDF is modeled as a normal distribution considering a median value of 1.5 and 25% variance, and is compared to results without any increase in tumor lethality for densely ionizing radiation [16].

## Results

**Fig 1:**
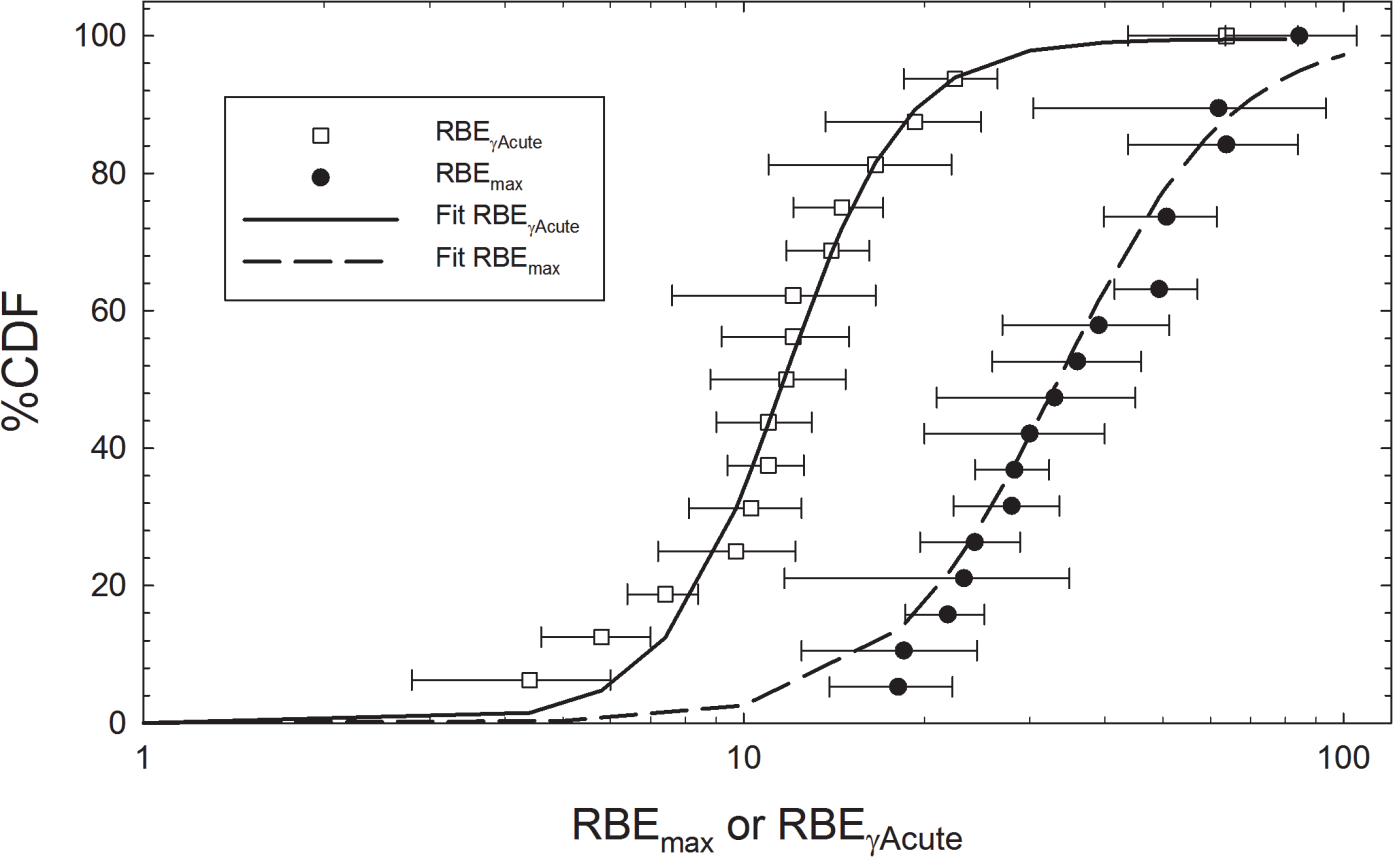
Cumulative distribution function (CDF) versus the maximum relative biological effectiveness (RBE) factor, RBE_max_ or RBE_γAcute_ based on experimental data from Table 2, and non-linear regression fits of the 3 parameter logistic equation to these data.

The 3 parameter logistics function provided a good fit to the CDF for the experimental RBE data for HZE particles and fission neutrons (Table 3; Fig. 1). Table 3 shows the estimate of the parameter Ʃ_0_/*α*_*γ*_ for the different models considered, and the mean values of RBE’s obtained. The large differences found between the different models suggest that understanding the impact of the apparent correlation whereby high DDREF values are often associated with the tumors with the highest RBE_max_ values is a critical factor for improving space radiation risk assessments.

**Table 3:** Parametric description for the cumulative distribution function (CDF) describing the uncertainty in maximum values of the relative biological effectiveness (RBE) factor.

Data Set	Ʃ_0_/α*_γ_* μm^2^ Gy	Mean RBE	R_0_	A	B
RBE_max_	11,370/6.24	40.0±19.5	33.7±1.9	1.01±0.056	−3.0±0.34
RBE_γAcute_	2,700/6.24	15.4±13.7	11.7±0.32	0.996±0.04	−4.28±0.51

Mean RBE factor values and standard deviations (SD), and values of parameters with SDs that result from fits of the logistic equation, CDF = A/(1+(RBE/R_0_)^B^) to distributions of RBEs from mouse experiments of Table 2 for RBE_max_ and RBE_γAcute_. Also shown is the central estimate of the Ʃ_0_/*α*_*γ*_ parameter found after considering the application of the quality factor (QF) function of Equation (4) or (8) to the average RBE from charged particles produced by fission neutrons or Fe particles (as described in the text).

**Fig 2:**
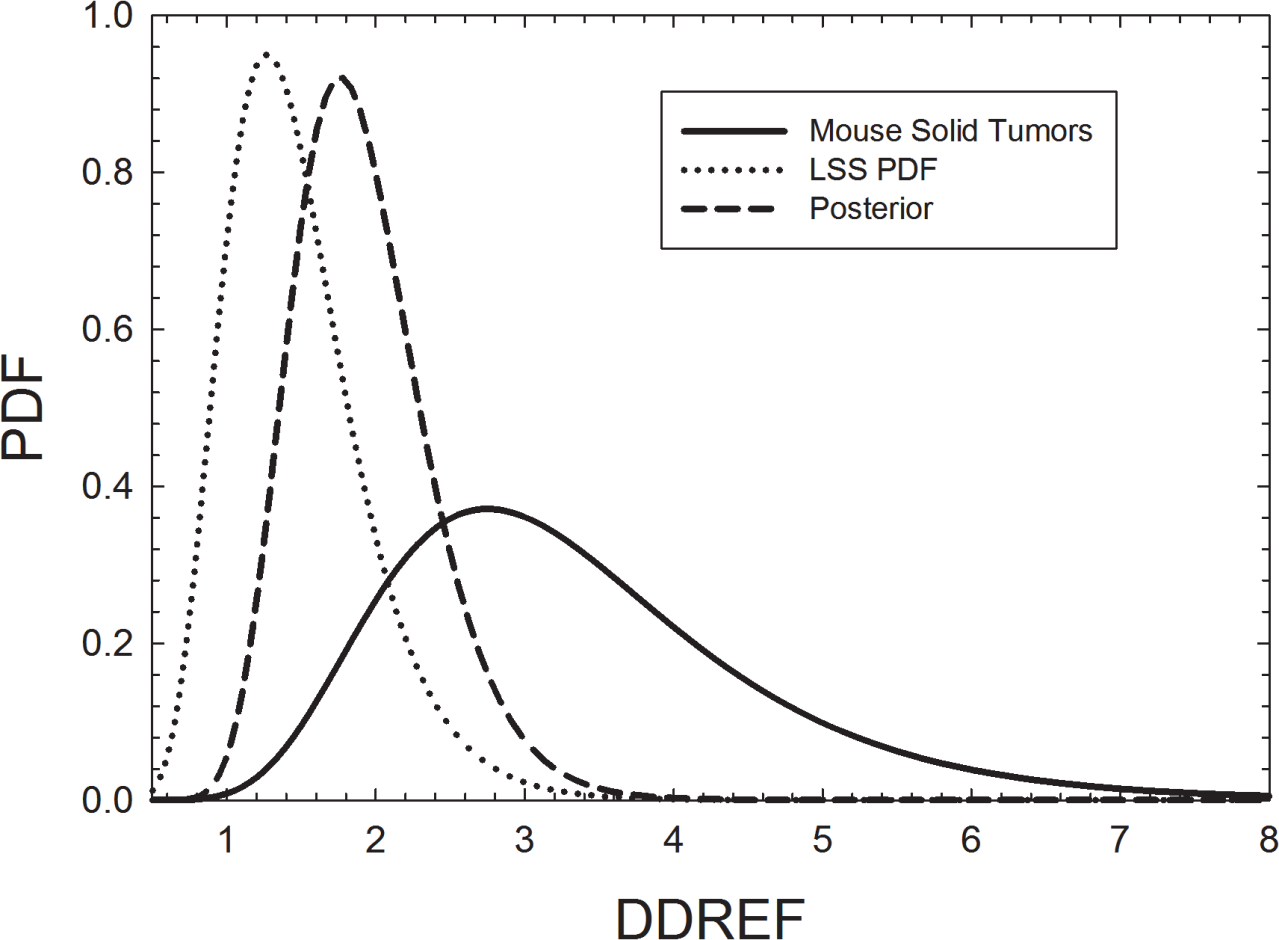
Bayesian analysis of the probability distribution function (PDF) representing the uncertainty in the dose and dose-rate reduction effectiveness (DDREF) parameter. The prior distribution is based on the BEIR VII report analysis of the Japanese survivor solid cancer data [30], the likelihood function uses the log-normal function to represent DDREFs for the mouse solid tumor data in **Table 2**, and the posterior distribution the prediction using Bayes theorem.

Fig. 2 shows results for the Bayesian analysis for the uncertainty in the DDREF parameter. The BEIR VII estimate [20] for the Japanese LSS study of DDREF = 1.3 with 95% confidence intervals (CI) of [0.8, 1.9] was used as the prior distribution, which is updated using Bayes theorem with the likelihood function for the RBE’s represented by a log-normal distribution. The resulting posterior distribution has a mean value of 1.88 with 95% CI of [1.18, 3.0]. For the central values of REID estimates for space missions discussed below we continue to use the value DDREF = 1.5 as recommended by the BEIR VII report and the review of the NSCR-2012 model by the NRC [6], however the posterior distribution is used to represent the PDF for the DDREF uncertainty in the analysis described here.

**Fig 3:**
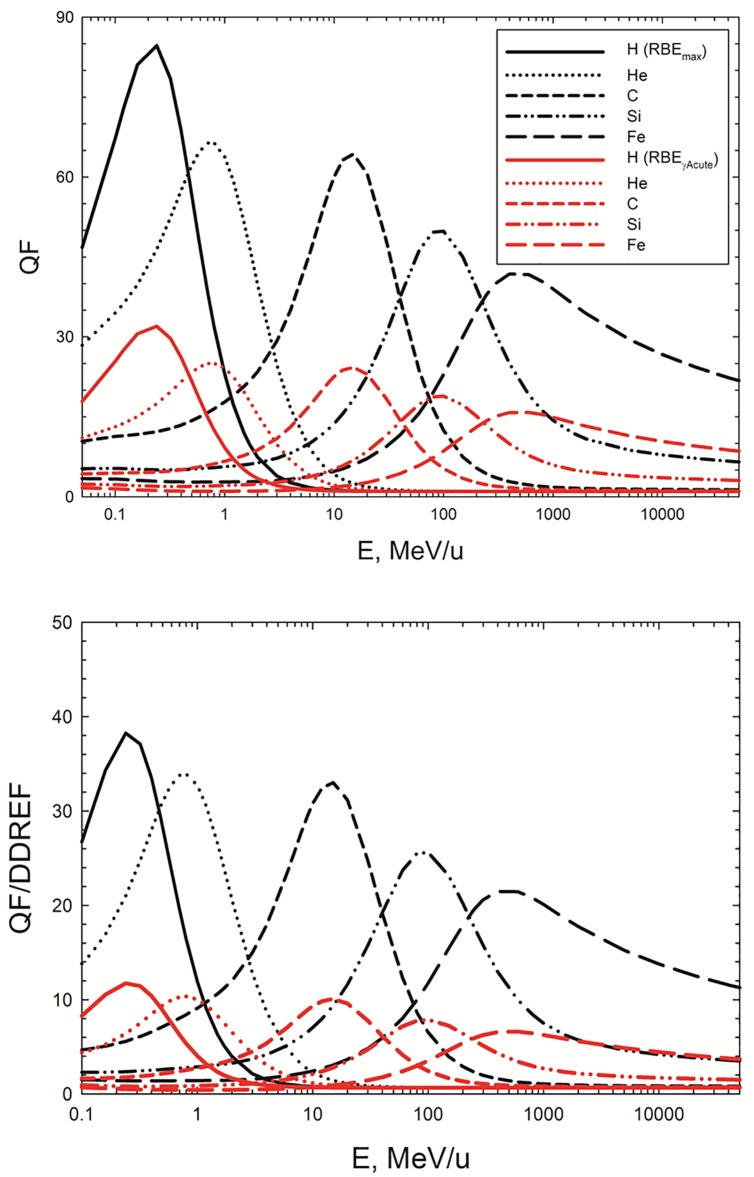
The quality factor (QF) or the ratio of the QF to the dose and dose-rate reduction effectiveness factor (DDREF), QF/DDREF versus the kinetic energy for several particle types. For the DDREF the recommended value of 1.5 [30] has been applied.

Fig. 3 shows values of the QF and QF_γAcute_ (upper panel) or of the ratios QF/DDREF and QF_γAcute_/DDREF (lower panel) versus E for H, He, C, Si, and Fe particles. These results using the value DDREF = 1.5 [30] suggest a significantly lower REID estimate will occur using the revised QF approach combined with an estimate of Ʃ_0_/*α*_*γ*_ directly from RBE and DDREF estimates from mouse solid tumor induction studies. The two approaches will become more similar if the DDREF value is increased. An approach that considers matched RBE and DDREF inputs into the REID estimates avoids some of the impact of estimates that ignore their possible correlation, however results in the use of two parameters derived from experiments compared to the RBE_max_ approach where the DDREF is estimated to a large extent from human data [30].

I next used the revised methods to make predictions for one-year missions in deep space for GCR particles representing the most recent solar minimum of 2009, which is noted to be somewhat more intense (less solar modulation) compared other recent solar cycles [10,11]. Table 4 shows predictions in the different models for 45-year old male and female never-smokers assuming a heavily shielded spacecraft (20 g/cm^2^ aluminum). A large reduction in the REID estimates and upper 90% or 95% CI’s occurs of about 40% using the QF_γAcute_ approach of Equation (8) compared to the use of Equation (4). Results using QF_γAcute_ are about 25% smaller in comparison to the previous NSCR-2012 model updated with the DevCan software from the CDC U.S. population rates, which has an improved representation of rates at older age [32]. Corrections to these rates for never-smokers were implemented in the same manner as in a recent report [10]. I also found in the final comparison of Table 4 that uses a previous approach [16] to consider the sensitivity to an increased tumor lethality for HZE particles and neutrons due to a qualitative difference not described by RBEs for tumor incidence, would negate a good portion of the reduction found using the revised QF model.

**Table 4:** Predictions of percentage risk of exposure induced death (%REID) for 1-year space missions at deep solar minimum.

Model	%REID	90% CI	95% CI
**Male Never-Smokers**			
NSCR*-2012	1.54	[0.57, 4.45]	[0.47, 5.85]
RBE_max_	2.05	[0.56, 5.68]	[0.47, 7.58]
RBE_γAcute_	1.2	[0.46, 2.87]	[0.39, 3.76]
RBE_γAcute_ with increased high LET** tumor lethality	1.43	[0.62, 5.03]	[0.52, 6.59]
**Female Never-Smokers**			
NSCR-2012	2.04	[0.64, 6.05]	[0.47, 7.86]
RBE_max_	2.75	[0.64,7.68]	[0.47, 9.95]
RBE_γAcute_	1.55	[0.51, 3.81]	[0.38, 4.99]
RBE_γAcute_ with increased high LET tumor lethality	1.89	[0.72, 6.9]	[0.52, 9.0]

Predictions of different models for %REID, and 90% or 95% confidence intervals (CI) for 45-y old male and female never-smokers on a 1-year space mission using the 2009 solar minimum galactic cosmic ray environment assuming 20 g/cm^2^ aluminum spacecraft shielding.

*NSCR is NASA Space Cancer Risk, RBE is relative biological effectiveness factor

**LET is linear energy transfer.

## Discussion

Because of the lack of human data on cancer risk following exposure to high LET radiation, animal studies continue to be the main source of information for risk estimates. Experimental models representing human cancer risks should be used to estimate the most meaningful RBE for risk assessment. The relevance of experimental models for human risks should be based on a small number of criteria [42,43] including: Models should represent tissues of interest for human risk, the cell of origin for cancer risks in humans, and possible mechanisms of cancer risk should be addressed. Also host factors that modify the expression of cancer in humans must be addressed to improve confidence in the use of experimental data for radiation risk predictions.

Recent studies are making efforts to develop appropriate mouse models of human risk using genetically engineered mice and other techniques [40,41,44], however they have not been used to estimate RBE values at this time. An important source of data on values of RBE or RBE_max_ for high LET radiation is tumor induction and life-shortening studies in mice or rats with HZE particles or fission neutrons (reviewed in [45,46]). Fission neutrons with energies from below 0.1 to about 5 MeV were found to be one of the most biological effective radiation fields, which can be attributed to the large biological effectiveness of low energy protons produced by neutrons at these energies. RBE estimates for tumor induction for the most biologically effective HZE particles such as high energy Fe particles are similar to ones found for fission neutrons, however very few studies of RBE’s for HZE particles have been reported.

The current study focused on published values of RBE’s for epithelial tumors in mice to make a new assessment of a key parameter in the NSCR model. RBE data for life-shortening in mice with fission neutrons is also available, however includes contributions from leukemia, thymic lymphoma, and connective tissue tumors, which typically have lower RBE’s compared to epithelial or solid tumors [35]. Mortality contributions from non-cancer diseases would also contribute to RBE estimates derived from life-shortening data. The direct application of RBE estimates for solid tumors made here avoids possible differences in RBEs for various endpoints. The resulting CDF’s for RBE values shown in Fig. 1 reflect variations of RBE with tissue type, sex, and strains of mice. It is not clear if a subset of these data would better reflect human solid cancer risks, while the limited amount of data severely limits such investigations.

The revised approach of Equation (8) considers the particle track structure to be made-up of low and high ionization density regions corresponding roughly to the particle penumbra and core, respectively. The biological effects of the high ionization density contribution (second term on the right-hand side of Equation (8)) of a particle track is assumed to be independent of dose-rate. However, the low ionization density contribution (first term on the right-hand side of Equation (8)), which is estimated from experiments at higher doses and dose-rates than would occur in space, is assumed to be dependent on dose-rate and is reduced by the DDREF for space mission risk predictions. This approach ignores NTE’s [13–15,47] and a possible inverse dose-rate effect that was observed for mouse tumor induction studies with fission neutrons at higher doses (>0.2 Gy) (reviewed in [45,46]). It is important that further studies of NTE’s and inverse dose-rate effects using HZE particles at space relevant doses (<0.2 Gy) be carried out.

I also have assumed that experiments for Fe particles and fission neutrons when used to estimate the maximum RBE for any radiation type are reflective of the same distribution. That RBE values for these distinct radiation types would be similar is suggested by the NASA QF biophysical model where fission neutrons produce proton spectra with reduced effectiveness compared to the most effective proton energy, while Fe particles at the optimal energy are less carcinogenic per unit dose compared to lighter charged particles of a distinct optimal energy. The variation that may occur due to the differences in effectiveness between fission neutrons and Fe particles is estimated to be less than 25% based on track structure and microscopic energy deposition considerations, but could be larger if distinct mechanisms of tumor induction occur for these distinct types of high LET radiation.

Cellular studies for chromosomal aberrations or gene mutations report similar RBE values found here with values near 10 to 25 found for HPRT mutations, so-called neoplastic cell transformation, and chromosomal aberrations (reviewed in [10]) relative to acute γ-ray doses, and higher values for RBE_max_. However, data for these endpoints lack scientific consensus for their use to represent tissue diseases such as cancer. Cellular endpoints were used for estimates of the shape parameters, *m* and *κ*, of the NASA QF due to small amount of data for tumor induction with different radiation types. The previous analysis [13] of the Harderian gland tumor induction studies considered 7 different particles [24, 25], and is found to have a high degree of similarity with the radiation quality dependence found for chromosomal aberrations.

The results of Table 4 for 1-year missions in deep space suggest that significant reductions in REID estimates and uncertainty bands are possible through improved understanding of dose-rate and radiation quality effects. Reductions using the QF_γAcute_ model of about 40% relative to the QF model using parameters derived from RBE_max_ from the same experiments for epithelial tumors in mice. Also reductions using the revised approach of about 25% relative to previous estimates [10, 11, 16] were found, with larger reductions of about 35% in upper 90% or 95% confidence levels.

Several reports [25, 28, 39–41, 44] have suggested that HZE particles and neutrons could produce more lethal tumors compared to tumors produced by low LET radiation or background tumors, which is a qualitative difference not accounted for in current risk estimates. For low LET radiation there is an implicit assumption made by epidemiology models that the tumors induced by radiation are similar to background tumors in a population. This assumption is consistent with the multiplicative risk model, and also based on lack of information to make an alternative assumption. Using the sensitivity analysis method described in a recent report [16] suggests that increases in tumor lethality for HZE particle and neutrons compared to background or low LET tumors as suggested by animal studies could substantially increase REID and uncertainty estimates negating the uncertainty reduction found here using a revised QF formalism, and therefore the radiation quality dependence of tumor lethality warrants further study.

## Conclusions

In the present work, RBE values for epithelial tumors in mice were used to assess a key parameter in the NASA QF model for solid cancer risk. This new approach makes better use of existing radiobiology data and avoids some of the subjectivity noted [6] in earlier estimates [10]. The revised model using QF’s based on acute exposures to γ-rays was shown to reduce REID predictions for space missions by about 25% compared to previous estimates [10, 11], and reductions in upper 90% or 95% CIs of about 35%.

An important feature of the revised QF model is its avoidance of comparisons to chronic exposures to γ-rays in space radiobiology studies. However estimates of DDREFs for low LET space radiation components, such as protons, mesons, and relativistic heavy ions of moderate charge number (Z<10) are still needed, and should be made directly with these radiation types in important experimental models.

There is still a vital need to obtain dose response data in mice for estimating RBE’s for a significant number of HZE particle types in several tissues including lung, mammary and digestive tumors where no RBE estimates have been reported. The limited information available suggests qualitative differences may occur in the metastatic potential of mouse tumors produced by different radiation qualities [28, 35, 41, 44]. This later feature of HZE particle cancer risk was estimated as a large uncertainty for space radiation risk estimates [16], and calculations reported here agree with this conclusion. It is to be noted that studies of low dose responses needed for RBE estimates and for elucidating potential qualitative differences in tumor types are not mutually exclusive and can be pursued in a harmonious manner with common research studies.
